# Enhancing the Corrosion Resistance of Austenitic Steel Using Active Screen Plasma Nitriding and Nitrocarburising

**DOI:** 10.3390/ma14123320

**Published:** 2021-06-15

**Authors:** Tomasz Borowski

**Affiliations:** Division of Surface Engineering, Faculty of Materials Science and Engineering, Warsaw University of Technology, 02-507 Warsaw, Poland; tomasz.borowski@pw.edu.pl

**Keywords:** S-phase, nitriding, nitrocarburising, pitting corrosion, microstructure

## Abstract

AISI 316L steel was subjected to active screen plasma nitriding and nitrocarburising. The processes were carried out at 440 °C for 6 h. The nitriding process employed an atmosphere of nitrogen and hydrogen, while nitrocarburising was carried out in nitrogen, hydrogen and methane. The processes yielded structures consisting of nitrogen and nitro-carbon expanded austenite, respectively. Microhardness was measured via the Vickers method, surface roughness using an optical profilometer, microstructure by means of light microscopy, while a scanning electron microscope (SEM) served to determine surface topography. Phase composition, lattice parameter and lattice deformation tests were carried out using the X-ray diffraction (XRD) method. Corrosion resistance measurements were performed in a 0.5 M NaCl solution using the potentiodynamic method. The produced layers showed very high resistance to pitting corrosion, while the pitting potential reached 1.5 V, a value that has not yet been recorded in a chloride environment. After the passive layer was broken down, there was a clear deceleration of pitting in the nitrocarburised layer. It was found that in the case of nitro-carbon expanded austenite, pits are formed much slower compared to the nitrogen austenite layer.

## 1. Introduction

Austenitic steels are very popular metallic materials used in the chemical, food, automotive, nuclear and medical industries, to name a few [1]. The corrosion resistance of austenitic alloys results from their high chromium content and the single-phase structure of paramagnetic austenite. The right content of chromium and molybdenum as well as the heterogeneity and stability of austenite contributes to increased durability of the passive layer [1]. Apart from the aforementioned elements, the austenite structure is further stabilised by light elements, which include nitrogen and carbon [2,3]. Recently, superaustenitic steels have drawn increased attention. Their composition includes nitrogen in amounts of several tenths of a percent and an increased chromium content compared to classic austenitic steels [4,5]. Superaustenitic steels have found use in the energy, paper and petrochemical industries [6]. These alloys are characterised by increased mechanical parameters, and in most cases increased tensile strength and yield strength as compared to conventional austenitic steels [4]. Superaustenitic steels produce values of resistance to pitting corrosion that clearly exceed those of commonly used austenitic steels, which are not stabilised by nitrogen [5]. Similar or even better corrosion resistance results can be obtained by the application of low-temperature nitriding (<450 °C) [7,8,9,10,11,12], carburising (<500 °C) [13,14,15] or nitrocarburising (<450 °C) [16,17,18,19] of austenitic steels that do not contain significant additions of nitrogen in their structure, such as, for example, AISI 304L, AISI 316L or AISI 321 steel. In these processes, the face centred cubic structure (fcc), which is present in the steel’s surface layer, shows a certain degree of deformability, which is dependent on the concentration of nitrogen and/or carbon [17,20]. The supersaturation of austenite with these elements leads to high-stress values in the layer and the distortion of the crystalline cell unit by up to 10% [21]. This process is accompanied by an increase in the stacking fault density of atoms in a plane (111) [2,20,21]. A diffusive surface layer with an addition of nitrogen and/or carbon in amounts exceeding its solubility in austenite, leads to the formation of expanded austenite known in the literature as the S-phase [2,22]. In addition to high corrosion resistance, hardness and wear resistance [23,24], the produced layer may also exhibit good biological properties, including antibacterial capabilities [25]. These enhanced properties mainly result from the higher stability of austenite achieved by introducing nitrogen and/or carbon atoms into the interstitial sites of the crystalline lattice [20,22].

Traditional diffusive processes incorporating plasma sputtering or ion implantation processes increase the surface roughness of steel and lead to a significant increase in compressive stress and structural defectiveness. Moreover, they usually yield only slight improvements in corrosion resistance, and at times even cause it to deteriorate [7,26,27]. Processes carried out under glow discharge conditions in low-temperature plasma, where the treated components are electrically isolated from the power source and placed inside a so-called active screen (the cathode), bring about much more desirable results [28,29,30]. The resulting layers display lower surface roughness and lack edge effect imperfections, as opposed to conventional glow discharge processes. These improvements are primarily due to a reduction of the cathodic sputtering effect. Low-temperature plasma nitriding or carburising are some of the better known austenitic steel surface hardening processes that make it possible to ensure a good level of corrosion resistance [7,8,13]. Carburised layers, unlike nitrided layers, are characterised by a gradient structure and demonstrate lower hardness and higher ductility [2,15,31]. The use of nitriding combined with carburising opens up new opportunities, including the possibility of obtaining thicker and more heterogeneous layers with a higher gradient [17,32], unobserved in the case of nitrided layers. It is also worth noting that the available literature lacks data concerning the influence of carbon co-occurring with nitrogen in the diffusion layer on the pitting corrosion of austenitic steels.

The aim of the study was to develop a process carried out at plasma potential in a temperature of 440 °C using an active screen and a determined mixture of nitrogen and hydrogen with or without methane, in order to improve the pitting corrosion resistance of AISI 316L steel. The study primarily aimed to analyse the impact of carbon in the presence of nitrogen on the corrosion properties of the steel.

## 2. Materials and Methods

### 2.1. Specimen Preparation

The investigations were conducted on AISI 316L austenitic steel with the following chemical composition in wt.%: C < 0.03, Si < 0.08, Mn < 2, P < 0.045, S < 0.03, Cr 16–18, Mo 2–2.5, Ni 12–15, the rest being Fe. The flat surfaces of cylindrically shaped ɸ 25 mm × 3 mm samples were ground using 240 to 800-grit SiC sandpapers (Lam Plan, Gaillard, France) and then cleaned in an ultrasonic cleaner (Intersonic, Olsztyn, Poland) with acetone (Chempur, Piekary Śląskie, Poland). The prepared samples were subjected to glow discharge nitriding and nitrocarburising at the plasma potential and were electrically isolated and placed inside an active, perforated screen made of AISI 304 steel. The processes were conducted via a semi-industrial device produced by the Institute of Precision Mechanics in Warsaw, Poland. The treatment device was described elsewhere [33].

Active screen plasma nitriding (ASPN) and nitrocarburising (ASPNC) were carried out at 440 °C for 6 h at a working chamber pressure of 100 Pa, while the working mixture composition in the case of nitriding was as follows: N_2_ and H_2_ at a ratio of 1:3 (flow rates of 50 and 150 sccm, respectively), while in the case of nitrocarburising: N_2_ and H_2_ at a ratio of 1:3 and CH_4_, which made up 5% of the entire gas mixture (at flow rates of 47, 143, 10 sccm, respectively). In the case of nitriding a voltage of 857 V and a current of 1.51 A were applied, while for nitrocarburising these values amounted to 898 V and 1.31 A, respectively. The temperature of the processes was monitored by a thermocouple, which was placed on an insulated table. Before treatment, the active screen used in this study was ASPN treated for 3 h with the same parameters mentioned before, in order to remove any contamination and oxide layers on its surface.

### 2.2. Microstructural and Hardness Analysis

The surfaces of the samples prepared for microscopic analysis were polished along the cross-sections of the layers using SiC abrasive papers up to 1200 grit and then with a 1 μm diamond suspension (Lam Plan, Gaillard, France). Etching of AISI 316L steel was carried out using a reagent consisting of 50% HCl + 25% HNO_3_ + 25% H_2_O (HCl and HNO_3_ produced by Chempur, Piekary Śląskie, Poland). The microstructures were imaged using a Nikon Eclypse LV150N optical microscope (Nikon Instruments Inc., Melville, NY, USA). Vickers microhardness was measured on the surface of the layers under a load of 50 g (HV0.05) using a Zwick tester (ZwickRoell GmbH & Co. KG, Ulm, Germany). The averaged results were obtained from at least 5 measurements taken for each sample.

### 2.3. Surface Topography Analysis

The roughness of the surface layer was analysed by means of a Wyko NT9300 optical profilometer (Veeco, Plainview, NY, USA). The measurements were carried out in at least 3 areas (470 µm × 627 µm) on the surface of each sample variant. The surfaces of the samples before and after nitriding and nitrocarburising were also observed with a Hitachi S-3500N scanning electron microscope (SEM, Hitachi, Tokyo, Japan).

### 2.4. Phase Composition Analysis

X-ray diffraction (XRD) analysis was carried out using a Bruker D8 Advance X-ray diffractometer (Bruker AXS GmbH, Karlsruhe, Germany) via CuK_α_ filtered radiation (*λ* = 0.154056 nm) at room temperature. The recording conditions were as follows: voltage 40 kV, current 40 mA, 2θ angular range from 30° to 60°, step Δ2θ—0.05°, count time—3 s. The recorded diffraction patterns were analysed using Brucker’s Diffrac.EVA.V.3.0 software (Bruker AXS GmbH, Karlsruhe, Germany).

### 2.5. Corrosion Measurements

The corrosion resistance of AISI 316L steel at an initial state and after ASPN and ASPNC was measured in a solution of 0.5 M (2.9%) sodium chloride (Chempur, Piekary Śląskie, Poland) with a pH of 7 by means of the potentiodynamic method using an Atlas-Sollich 0531 EU&IA device (Atlas-Sollich, Rębiechowo, Poland). A three-electrode setup was used in the tests comprising a test electrode, a saturated calomel electrode (SCE, Eurosensor, Gliwice, Poland), i.e., the reference electrode and a platinum gauze as a counter electrode. Before the tests, the samples were kept in the measurement array for 2 h to stabilise open circuit potential (OCP). Afterward, the polarisation resistance R_pol_ was gained via the Stern method by polarising the test material from a potential of 10 mV lower to 10 mV higher than the determined OCP at a sweep rate of 0.2 mV·s^−1^. The polarisation resistance was determined on the basis of the E = f(i) dependence. The anodic polarisation curves of the tested materials were then registered via the potentiodynamic method. The samples were polarised from a potential 0.2 V lower than the OCP, to a potential of 1.5 and 2 V. In the potential range of ±200 mV from the OCP, a polarisation rate of 0.2 mV·s^−1^ was used, whereas in the remaining potential range the rate was 0.8 mV/s. Pitting potentials E_pit_ and the anode current densities in the passive layer range i_pas_ were evaluated from the polarisation curves. The corrosion current density i_corr_ and corrosion potential E_corr_ were determined using the Tafel extrapolation method. All of the corrosion tests were performed three times to verify reproducibility. After potentiodynamic tests, the samples’ surfaces were observed again using a Hitachi S-3500N SEM (Hitachi, Tokyo, Japan).

## 3. Results and Discussion

**Figure 1:**
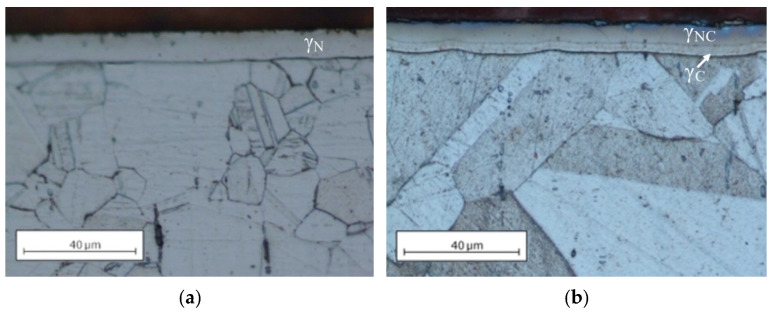
Microstructure of the ASPN (active screen plasma nitriding) (**a**) and ASPNC (nitrocarburising) (**b**) layers on AISI 316L steel.

**Figure 2:**
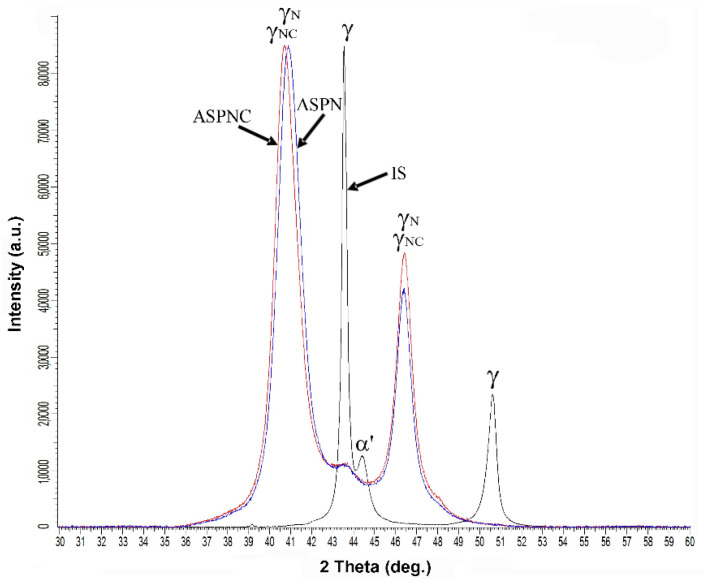
XRD patterns for AISI 316L austenitic steel in its initial state (IS) and after ASPN and ASPNC; γ—austenite, γ_N_—nitrogen S-phase, γ_NC_—nitro-carbon S-phase, α′—deformation martensite.

Figure 1 presents the microstructures of the nitrided and nitrocarburised layer. The layer formed in this temperature consisted of expanded austenite, which is evidenced by XRD examinations (Figure 2). In the case of the nitrided layer, a homogeneous structure of γ_N_ can be observed with a clear, sharp front approx. 9.7 μm deep (Figure 1a). In turn, the nitrocarburised layer showed a dual-layer structure comprising two layers of γ_NC_ and γ_C_ separated by a clear border, with a total thickness of 10.5 μm, where γ_NC_ measured 7 μm, while γ_C_–3.5 μm (Figure 1b). The outer, larger part of the layer consisted mainly of nitrogen S-phase while on the inside it was mainly softer carbon S-phase (Figure 2), making it thus a gradient structure.

This structure type results from the differences in the diffusion coefficients of carbon and nitrogen within austenite. Due to the smaller atomic diameter and a lower affinity to chromium and iron, carbon diffuses more easily in the surface layer of AISI 316L steel than nitrogen. This structural model is also commonly found among the diffusion layers of austenitic steels containing small amounts of carbon which are subjected to a bare form of nitriding, in the absence of carbon sources in the gas mixture. The mechanism by which the internal carbon S-phase layer is formed is referred to as the “pushing effect” of nitrogen on carbon [2,9,17,32,34,35,36]. Furthermore, in the area between the S-phase layer and the steel core, a very thin, dark zone appears. Its presence under the light microscope results from a difference in height between the hard layer and the softcore developed during the polishing of the metallographic specimens (Figure 1a,b). The XRD patterns shown in Figure 2 present austenite peaks of AISI 316L steel in its initial state as observed at a 2θ angle of about 43.5 and 50.5°. For this material, a martensite peak can also be observed at a 2θ angle of approx. 44.5°. This structure was formed on the steel surface during sample preparation. As a result of the pressure applied on the sample surface during polishing, a plastic deformation-induced martensitic transformation occurred (TRIP effect), a phenomenon also reported by researchers in other studies [37]. In the case of layers nitrided and nitrocarburised at a temperature of 440 °C, expanded peaks were observed, which corresponded to nitrogen S-phase (γ_N_) or nitro-carbon S-phase (γ_NC_), respectively. Deformation martensite α′ was not observed in these layers. This is due to reverse transformation taking place. In the process, a solid solution of nitrogen and/or carbon in the deformation martensite (α′_N_ or α′_C_) is formed. A decrease in the austenitic transformation start temperature A_s_ is observed as the concentration of nitrogen and/or carbon in the structure increases. At a certain concentration of nitrogen and/or carbon, the A_s_ temperature reaches a value equal to the process temperature (440 °C) resulting in the transformation of the α′_N_ nitrogen martensite or α′_C_ carbon martensite to reversed nitrogen S-phase (γ_N_) or carbon S-phase (γ_C_). A similar mechanism was observed during the nitriding process of metastable chromium-free, high-nickel austenitic steel [37] and AISI 304 steel [38] previously subjected to martensitic transformations. In addition, the layers did not contain the CrN phase, the presence of which contributes to the segregation of chromium in the austenitic structure in the proximate grain-boundary locations and causes intergranular corrosion [28]. Its absence is attributed to the application of a process temperature that did not exceed 450 °C.

The supersaturation of austenite with nitrogen and carbon leads to the expansion of the crystalline lattice, which is manifested by the increasing value of the lattice parameter. On the basis of the diffraction patterns (Figure 2), the lattice parameters *a* and its distortion *ε* in the ASPN and ASPNC layers were determined and compared to the γ initial-state austenite lattice (Table 1). Distortion was calculated according to the formula:

*ε* = Δ*a*/*a_γ_* (%) (1)

where Δ*a* = *a_γx_* − *a_γ_*; *a_γ_*—lattice parameter for austenite; *a_γx_*—lattice parameter for nitrogen and nitro-carbon expanded austenite, respectively.

The results show that the nitrocarburised layer has a slightly higher lattice parameter as compared to the nitrided layer, which in turn results in a greater expansion of the crystal lattice, i.e., 8.6 and 9.2%, respectively, for nitrogen and nitro-carbon S-phase. The nitrocarburised outer layer consists essentially of the S-phase supplemented with nitrogen, while the carbon that diffuses is mostly pushed into the deeper zone of the layer with a certain amount of carbon still remaining in the outer layer [31,32,34,35,36]. As mentioned previously, the use of Cu-Kα radiation makes it possible to analyse the surface layer of steel to a depth of approximately 4 μm, hence this measurement did not make it possible to analyse the carbon austenite (γ_C_) found in the deeper part of the double layer (Figure 1b). It can therefore be concluded that the carbon present in small quantities in the outer, first part of the nitrogen-enriched layer (γ_NC_) is responsible for the slight change in the lattice parameter and its expansion observed following ASPNC.

The nitrided and nitrocarburised layers formed were characterised by a hardness over 3 times greater than the hardness of the steel core (Table 2). The lower hardness of the nitrocarburised layer compared to the nitrided layer mainly resulted from the lower hardness of sublayer γ_C_ [31,32] and a lower thickness of layer γ_NC_ (Figure 1b) compared to layer γ_N_ (Figure 1a). The formed nitrided and nitrocarburised layers had a rougher surface than the surface of the steel in its initial state (Table 2). The Ra parameter increased more than twofold, however, when comparing the layers it was observed that their surface parameters did not differ much from each other.

**Figure 3:**
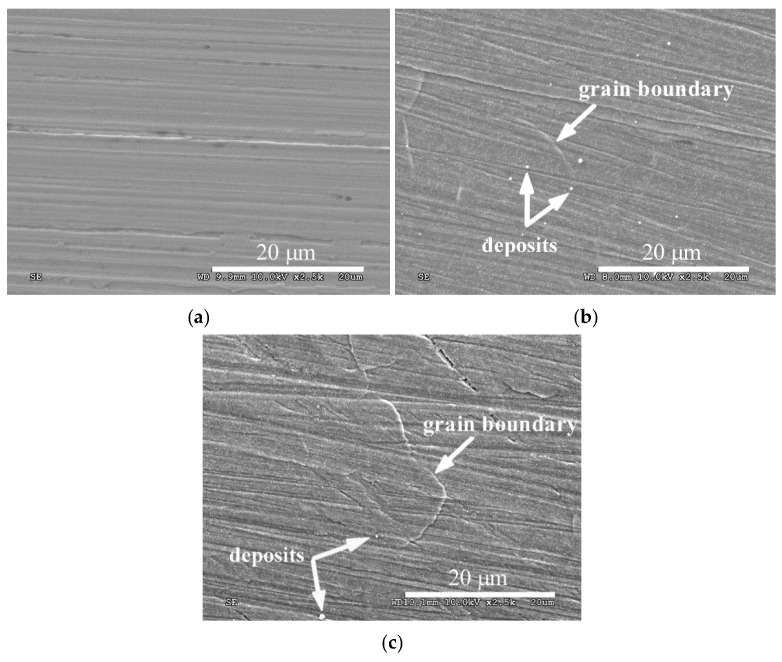
Surface morphology of AISI 316L steel in its initial state (**a**) and of ASPN (**b**) and ASPNC (**c**) layers.

Figure 3 shows the surfaces of austenitic steel in its initial state and after ASPN and ASPNC. An increase in surface development is observed following the glow discharge treatments. Changes in the topography of the steel surfaces stem mainly from the processes that occurred at the grain boundaries. During SEM observations, deformation in the vicinity of the boundaries was observed, which resulted from the significant stress that had accumulated during the anisotropic diffusive supersaturation of grains with different crystallographic orientations. This process led to different degrees of expansion of the neighbouring grains (Figure 3b,c) and manifested itself by a relief visible on the steel surface [39], which was not observed in the case of steel in its initial state (Figure 3a). The relief was more noticeable on the surface of the nitrocarburised layer, which can be associated with greater lattice expansion (Table 1) and the associated higher residual stress [17]. The changes observed at the grain boundaries could also be indicative of intercrystalline cracking in the S-phase, however, no propagation of cracks from the surface on the cross-section in the microscopic tests was observed. Very small deposits were also observed on the surface of the layers, which were applied to the sample surfaces by way of cathodic sputtering of the active screen (Figure 3b,c). To some extent, they also contributed to an increase in surface roughness after the ASPN and ASPNC processes. However, the particles did not form a compact, continuous layer of iron or chromium nitrides (Figure 1 and Figure 2), which could hinder the diffusion of nitrogen and carbon in the surface layer of steel [40,41].

**Figure 4:**
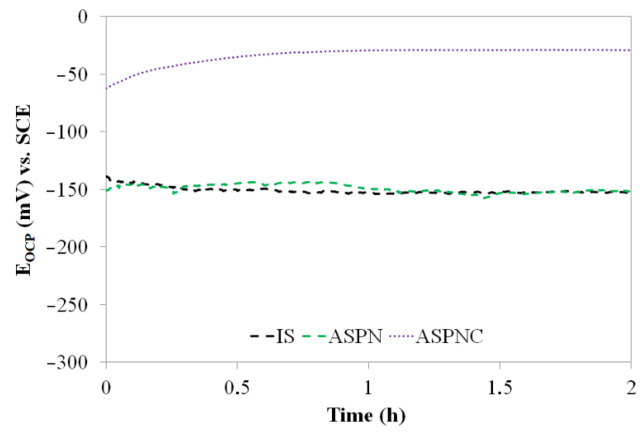
OCP (open circuit potential) curves of AISI 316L steel in its initial state (IS) and after ASPN and ASPNC; SCE—saturated calomel electrode.

Figure 4 displays the open circuit potential (OCP) of AISI 316L steel in its initial state and after ASPN and ASPNC. The stability of the passive film is closely correlated with the OCP. A more positive OCP value results in a more complete and durable passive film. It is observed that the nitrocarburised layer shows significantly more positive OCP values as compared to steel in its initial state and the ASPN layer. At the same time, the curve remains stable, which proves a more durable passive film. Curves for AISI 316L steel and the nitrided layer present much lower OCP values and they are not as stable as the ASPNC layer. The OCP for the ASPN layer and steel in its initial state present quite similar values.

**Figure 5:**
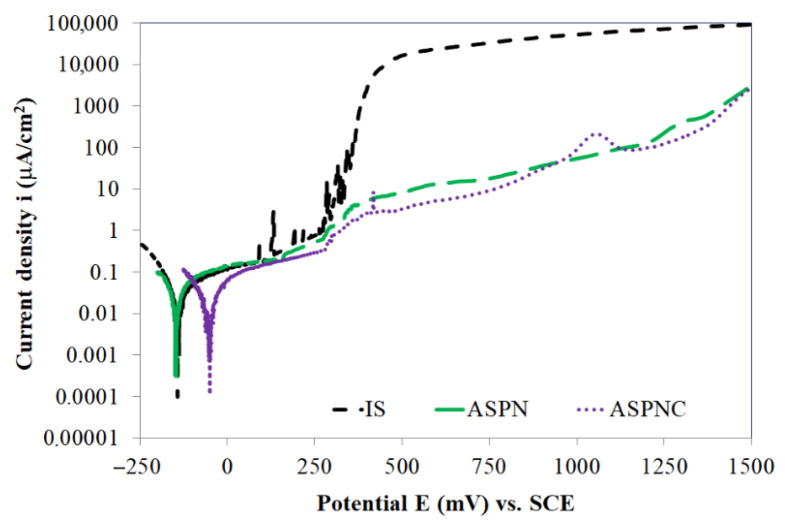
Anodic polarisation curves (up to 1500 mV) of AISI 316L steel in its initial state (IS) and after ASPN and ASPNC.

The anodic polarisation curves produced in an aqueous solution of 0.5 M (2.9%) NaCl prove that the layers composed of nitrogen and nitro-carbon expanded austenite have lower anodic current densities in a wide potential range of up to 1500 mV as compared to austenitic steel not subject to thermo-chemical treatment (Figure 5), which can be associated with a very good resistance of the layers to localised corrosion. The best corrosion properties were demonstrated by steel with a surface layer of nitro-carbon expanded austenite. The corrosion potential for this variant was −51 mV, which was almost three times as much as the results for steel in its initial state (−142 mV) and for glow discharge nitrided steel (−148 mV) (Table 3).

**Figure 6:**
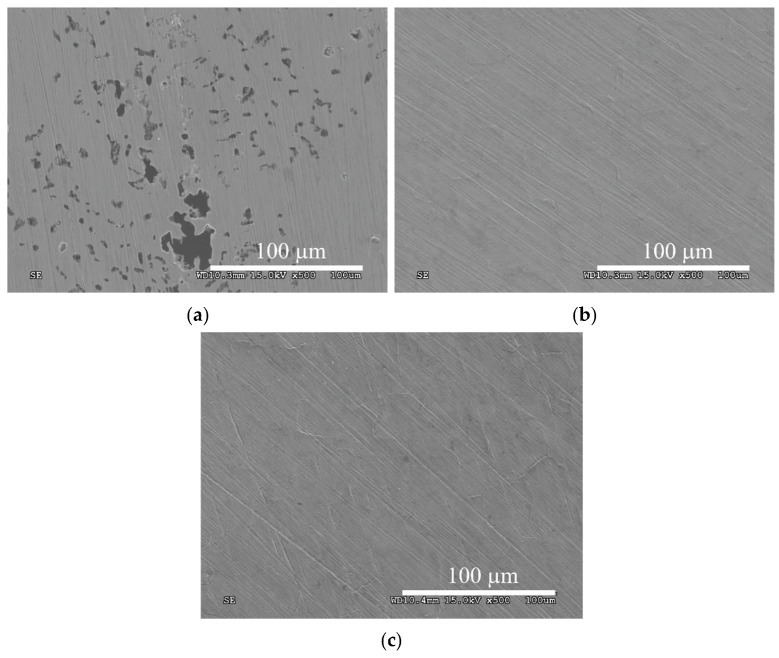
Images of the surface of AISI 316L steel in its initial state (**a**), following ASPN (**b**) and ASPNC (**c**) after potentiodynamic tests up to 1500 mV.

The corrosion current density for all variants was quite similar and ranged from 0.003 to 0.008 μA·cm^−2^. The ASPNC layer was characterised by the highest polarisation resistance R_pol_ of 766 kΩ·cm^2^, which was more than 250 kΩ·cm^2^ greater compared to the resistance of AISI 316L steel in its initial state and after the ASPN process. The presented E_corr_, i_corr_ and R_pol_ parameters define the resistance of austenitic steel to uniform corrosion but in the presence of Cl^−^ ions, the key parameters are passive state current density i_pas_ and the pitting potential E_pit_ defining the durability of the passive layer. The ASPNC layer demonstrated the lowest passive state i_pas_ current density of 9 μA·cm^−2^ measured at a potential of 750 mV, for the ASPN layer, this value amounted to 18 μA·cm^−2^. In turn, steel in the initial state underwent pitting corrosion at this potential (Table 3). Nitriding and nitrocarburising led to a significant increase in the pitting potential. No clear evidence of pits was observed on the surface of the tested layers in the entire measurement range of up to 1500 mV (Figure 5 and Figure 6b,c). On the other hand, in the case of steel in its initial state, pits formed throughout the greater part of the anodic range up to 1500 mV, which is evidenced by a sudden increase in anodic current density at the value of 355 mV (Figure 5 and Figure 6a). Chloride ions are capable of causing the passive oxide layer of stainless steel to break down locally, e.g., at inclusions, discontinuities and in mechanical flaws. Such areas are recognised as weak locations, which are more pervious to anion ingress and anodic dissolution [42].

It should be noted that a pitting potential greater than 1500 mV is very high, and so far has not been reported in the literature. The achieved E_pit_ potential values are all the more surprising in view of the fact that the tests were conducted in a 0.5 M NaCl solution with a concentration of Cl^−^ ions similar to that which is found in natural seawater. Cisquini et al. [19] investigated the influence of the roughness induced by sputtering during plasma nitrocarburising and of process temperature on the corrosion resistance of AISI 304 austenitic stainless steel in a 3.5% aqueous solution of sodium chloride. For example, a specimen nitrocarburised at 430 °C with a layer thickness of 12.9 µm and surface roughness of 0.71 µm showed a pitting potential of approx. 300 mV. Furthermore, it was found that an expanded nitrocarbon austenite layer, which was thinner and had lower surface roughness, featured a higher corrosion resistance value. In another study, Kajzer et al. [18] examined implants for treating deformations of the anterior chest wall made of AISI 316 LVM (Low Carbon, Vacuum Melt) steel, which were subjected to active screen nitriding, nitrocarburising (T = 420 °C, t = 60 min), sterilisation and exposed to Ringer’s solution at 37 °C. Such steel, compared to AISI 316L, exhibits better resistance to localised corrosion due to its slightly modified chemical composition and manufacturing process (vacuum melting). Thin layers of nitrogen (2 mm) and nitrocarbon (3.8 mm) S-phase showed high pitting potentials of up to 1400 mV. It was found that the best set of properties (hardness, resistance to pitting and crevice corrosion) after sterilisation and exposure to Ringer’s solution was displayed by implants with a diffusion nitrocarburised layer. Lei et al. [43] presented the results of potentiodynamic tests of AISI 316L after nitriding in electron-cyclotron resonance microwave plasma at a temperature of 380 °C, which made it possible to obtain the S-phase in the surface layer. The authors investigated steels and diffusion layers with a thickness of 12 μm in Ringer’s solution with a pH of 3.5–7.2 at 37 °C. In a solution with a pH of 7.2, the pitting potential for AISI 316L steel amounted to approx. 300 mV, while after ion nitriding, this potential reached a value of approx. 800 mV. The studies were carried out at room temperature, but it is worth noting that Ringer’s solution is less aggressive than a solution consisting of 0.5 M NaCl (approx. 20.6 g·L^−1^ lower concentration of NaCl). In turn, Baranowska et al. [8] investigated the corrosion resistance of austenitic stainless steel subjected to gas nitriding at various temperatures from 400 to 570 °C and at different ammonia concentrations. The studies were conducted in a 3% NaCl solution. Based on the presented curves it can be concluded that steel nitrided at 400 °C in an atmosphere of 100% ammonia presented the best corrosion resistance. The pitting potential achieved was a little more than 800 mV. A similar increase in resistance to pitting corrosion was observed in superaustenitic steel enriched with nitrogen throughout its entire volume range. Nagarajan et al. [5] in their studies used natural seawater collected near the town of Chennai, India. The concentration of Cl^−^ and Na^+^ in the water was similar to the concentration of ions in a 0.5 M NaCl solution. The study compared the corrosion resistance of AISI 316L austenitic steel and that of Alloy 33 and Alloy 24, the composition of which is significantly different from that of AISI 316L steel, especially in terms of their higher chromium, nickel and nitrogen content. In addition, Alloy 24 has higher concentrations of manganese. A significant increase in the potential at which crevice corrosion begins was observed (200 mV to approx. 800–900 mV), however, it is difficult to determine how large of an effect the nitrogen present in Alloy 33 and Alloy 24 had on corrosion resistance given the changes in the concentration of other important elements that affect corrosion resistance. Li et al. [28] studied AISI 316 steel that had been subjected to ASPN at 500 °C and 420 °C and tested in a 3.5% solution of NaCl. Following the process at the higher of the two temperatures given above, the researchers observed intergranular corrosion taking place on the surface of the layer, which developed as a result of the segregation of alloying elements in the steel and precipitation of, e.g., chromium nitride. Layers nitrided at 420 °C demonstrated better resistance to pitting corrosion and lower current densities in the anodic range. No pits on the surface of the layer consisting of the S-phase were observed, nevertheless, crevice corrosion was seen to occur along the edge of the tested area which came in contact with the gasket, manifesting itself by an increase in current densities at a potential value of approx. 1.2 V. Borgioli et al. [44] tested three types of austenitic steel: AISI 316L, A202 and nickel-free P558, all subjected to low-temperature plasma nitriding at 360 and 380 °C. Potentiodynamic tests were carried out in a 5% undeoxygenated NaCl solution and the obtained pitting potentials did not exceed 1.2 V. For all the examined steels these values were similar to each other.

**Figure 7:**
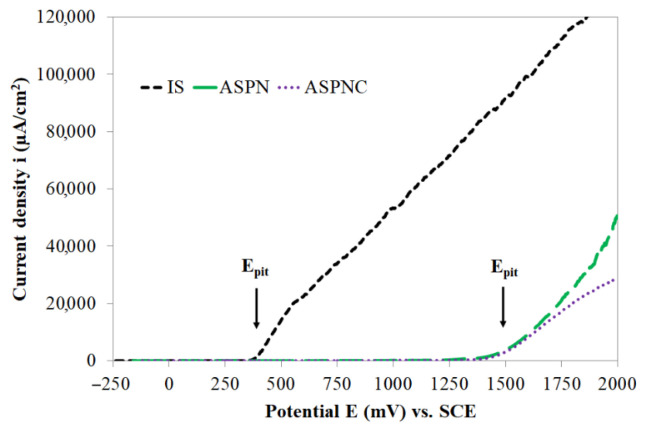
Anodic polarisation curves (up to 2000 mV) of AISI 316L steel in its initial state (IS), after ASPN and ASPNC.

**Figure 8:**
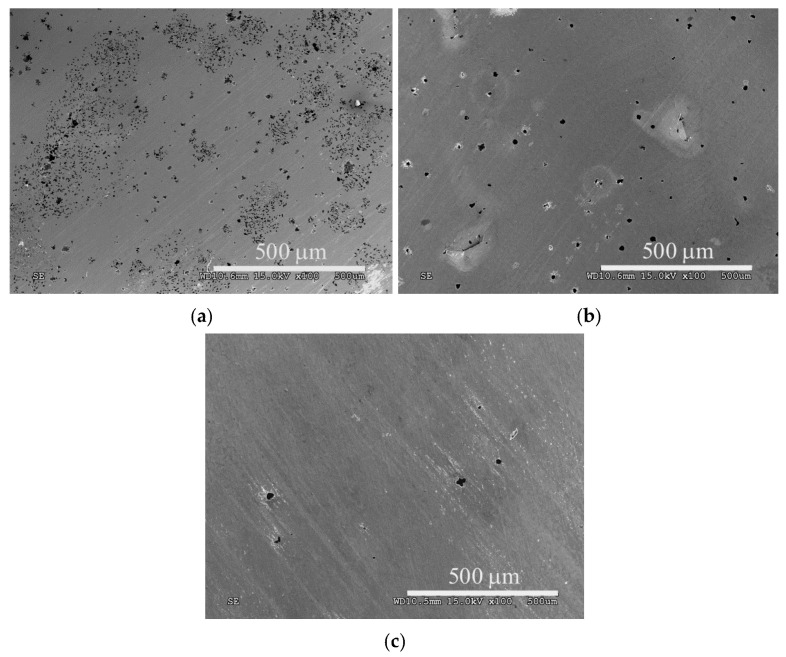
Images of the surface of AISI 316L steel in its initial state (**a**), following ASPN (**b**) and ASPNC (**c**) after potentiodynamic tests up to 2000 mV.

Potentiodynamic tests of steels resistant to corrosion are usually carried out to a value of 1000–1500 mV [5,8,32,43,45]. The tests conducted in this study in the range up to 1500 mV did not make it possible to determine pitting corrosion values for ASPN and ASPNC layers, therefore further potentiodynamic tests were performed, this time to a potential of 2000 mV (Figure 7). In order to make a more precise analysis of the change taking place on the surface of AISI 316L steel and the S-phases, the current density axis on the diagram was presented linearly. In the case of both layers, a clear increase in current densities is observed at a potential of approx. 1500 mV. It can be concluded that the produced layers demonstrated the same pitting corrosion value, nevertheless, the rates of pit formation of the tested layers was not identical. In the case of the nitrided layer, a greater increase in current density is observed after the pitting potential is exceeded. The current density value for the ASPN layer at a potential of 2000 mV was 5.05 × 10^4^ μA·cm^−2^, while for the ASPNC layer it was 2.86 × 10^4^ μA·cm^−2^. These results may be indicative of a higher level of stability of the layer composed of nitro-carbon expanded austenite as compared to the nitrogen expanded austenite layer, which in turn leads to an improvement of the passive layer being formed on the surface of the tested materials in the course of anodic polarisation. The results obtained on the basis of the polarisation curves confirm the layer surface observations performed using SEM (Figure 8).

A large number of small pits, which formed from a potential of 355 to 2000 mV, are observed on the surface of AISI 316L steel (Figure 8a). In the case of the ASPN layer, there were much fewer pits (Figure 8b) than on the surface of the steel in its initial state, which was due to a pitting potential close to 1500 mV. A significantly slower increase in corrosion current density following nitrocarburising suggests a slower expansion of the pits, as observed by the decreased number of pits formed after corrosion tests (Figure 8c) as compared to the nitrided layer (Figure 8b).

The use of a modified process carried out at the floating potential, incorporating an active screen made it possible to produce layers with significant corrosion resistance. The pile-ups and deformations visible at the grain boundaries (Figure 3b,c) that appeared after ASPN and ASPNC, most probably did not have a considerable impact on the continuity and durability of the passive layer formed on AISI 316L steel. The observed increase in corrosion resistance may have been chiefly caused by the chemical changes in the surface layer of the steel. Several possible mechanisms were proposed in the literature. One of them is based on a cathodic reaction that slows down the pitting corrosion rate:

[N] + 4H^+^ + 3e^−^→NH_4_^+^ (2)

Ammonium ions that form from the nitrogen contained in the S-phase lead to neutralisation of the acidic environment in the pits as a result of H^+^ ion consumption, which in turn leads to an increase in the pH level [43,46] and inhibition of the hydrogen evolution cathodic reaction:

2H^+^ + 2e^−^→H_2_↑ (3)

as well as equivalent anodic reactions, mainly the oxidation of iron:

Fe→Fe^2+^ + 2e^−^ (4)

and also other alloying elements contained in austenitic steel such as chromium, nickel, molybdenum.

The nitrogen present in austenitic steel also inhibits the precipitation of intermetallic phases such as σ, χ and also of M_23_C_6_ carbides, whose presence contributes to a lowering of corrosion resistance [6]. The enrichment of the austenitic steel surface with nitrogen may additionally contribute to its stabilisation and an increase in the durability of the passive layer, thanks to which it can more easily resist attacks by aggressive ions. Nitrate ions (NO_3_^−^) can also play a significant role in improving the resistance of the passive layer being formed against corrosive factors [28].

Based on the test results it is concluded that adding methane into the working atmosphere during nitrocarburising did not lead to any significant changes in the passive state as compared to the nitrided layer. However, once the E_pit_ pitting potential was exceeded, a slowing down of corrosion processes in the layer, in which carbon atoms are located interstitially beside nitrogen, was observed (Figure 7). The ASPNC layer is characterised by improved properties compared to the ASPN layer, which is confirmed by the significantly fewer pits visible in corrosion tests (Figure 8c) formed in the range from 1.5 to 2 V. It can be assumed that probably carbon atoms, such as nitrogen atoms released in the initial stage of the corrosion process, react with H^+^ ions to form methyl cations, which increase the pH in the pits, thus promoting repassivation:

[C] + 3H^+^ + 2e^−^→CH_3_^+^ (5)

## 4. Conclusions

Based on the presented analysis it can be concluded that:

1. Active screen plasma nitriding or nitrocarburising are technologies that guarantee a very large increase in the corrosion resistance of austenitic steels in an aggressive environment containing Cl^−^ ions. The use of active screen plasma processes makes it possible to produce an austenitic steel surface layer characterised by a significant increase in the durability of the passive layer over a broad range of potentials.
2. The pitting potentials for ASPN and ASPNC layers are similar, i.e., 1500 mV, however, the kinetics of pit formations are different in both cases. In the case of nitro-carbon expanded austenite, pits are formed much slower than in the case of the nitrogen austenite layer, which is indicative of the significant role that carbon, in combination with nitrogen, plays in inhibiting pitting corrosion processes.
3. The changes in properties that carbon contributed to also included increased layer thickness, a change in the microstructure (a double-layer structure consisting of γ_NC_ and γ_C_) and in the degree of hardness.
4. Nitrocarburising carried out using the proposed technology and parameters may help extend the scope of application of cheaper, conventional austenitic steels. Steels subjected to this type of treatment can be utilised in various sophisticated solutions, which typically involve the use of superaustenitic steels. These can be found, among others, in the medical, petrochemical and power industries, where high corrosion resistance of some structural elements is often crucial.

## Figures and Tables

**Table 1:** Lattice parameter *a* and lattice distortion *ε* in the surface layer of AISI 316L steel in its initial state (IS) and after ASPN and ASPNC.

Material	*a*, nm	*ε*, %
IS	0.360	-
ASPN	0.391	8.6
ASPNC	0.393	9.2

**Table 2:** Surface hardness (HV) and roughness (Ra) of AISI 316L steel in its initial state (IS) and following ASPN and ASPNC.

Material	HV0.05	Ra, nm
IS	264 ± 5	62 ± 3
ASPN	1207 ± 38	158 ± 3
ASPNC	972 ± 16	169 ± 1

**Table 3:** Electrochemical values of AISI 316L steel in its initial state (IS) and after ASPN and ASPNC.

Material	E_corr_, mV	i_corr_, μA·cm^−2^	R_pol_, kΩ·cm^2^	i_pas_, μA·cm^−2^ (at 750 mV)	E_pit_, mV
IS	−142	0.008	516	-	355
ASPN	−148	0.007	502	18	≥1500
ASPNC	−51	0.003	766	9	≥1500

E_corr_—corrosion potential; i_corr_—corrosion current density; R_pol_—polarisation resistance; i_pas_—passive state current density; E_pit_—pitting potential.

## Data Availability

All the data is available within the manuscript.
